# A Rare Presentation of Vulvar Discoid Lupus Erythematosus Masquerading as Lichen Sclerosus

**DOI:** 10.1155/crog/6697676

**Published:** 2026-04-17

**Authors:** Asiya Cummings, Joseph M. Maurice, Marylee Braniecki

**Affiliations:** ^1^ Department of Obstetrics and Gynecology, Advocate Illinois Masonic Medical Center, Chicago, Illinois, USA, advocatehealth.com; ^2^ Department of Obstetrics and Gynecology, University of Illinois Chicago College of Medicine, Chicago, Illinois, USA; ^3^ Department of Pathology, University of Illinois College of Medicine, Chicago, Illinois, USA, uic.edu

## Abstract

Discoid lupus erythematosus (DLE) is a chronic cutaneous manifestation of lupus that may occur independently or precede systemic disease. Vulvar involvement is exceptionally rare and can mimic more common vulvar dermatoses. We report a case of vulvar hypertrophic DLE in a 55‐year‐old African–American woman with systemic lupus erythematosus who was presented with an asymptomatic erythematous, ulcerated vulvar lesion initially suspected to represent herpes simplex virus infection or lichen sclerosus. Histopathologic examination demonstrated vacuolar interface dermatitis, follicular plugging, basement membrane thickening, and stromal mucin deposition, findings consistent with DLE. The lesion developed during a lupus flare and lacked the pruritus typical of lichen sclerosus. Management included continuation of immunosuppressive therapy with clinical improvement and residual postinflammatory hypopigmentation. This case highlights the importance of considering lupus‐related disease in the differential diagnosis of atypical vulvar lesions and underscores the role of biopsy in establishing an accurate diagnosis.

## 1. Introduction

Discoid lupus erythematosus (DLE) is a skin manifestation of lupus that can occur alone or before systemic symptoms. It is known for distinct red plaques, sometimes leading to scarring. Our case report demonstrates an exceptionally rare instance: DLE localized only to the vulva. Highlighting its features, diagnosis, and differentiation from similar conditions, this sheds light on a unique presentation and aids fellow gynecologists in accurate identification.

## 2. Case Presentation

A 55‐year‐old African American female with a history of systemic lupus erythematosus presented with an asymptomatic vulvar lesion noted during a lupus flare, characterized by generalized fatigue and weakness. She denied pruritus, pain, or ulcerations of other mucosal surfaces. Her medications included hydroxychloroquine 200 mg daily, which she had not taken for approximately 1 month, azathioprine 25 mg daily, and prednisone 20 mg daily.

Physical examination revealed a well‐demarcated erythematous, ulcerated, and white plaque‐like lesion involving the vulva. There was no associated tenderness or pruritus. The initial clinical differential diagnosis included herpes simplex virus infection and lichen sclerosus.

Laboratory evaluation demonstrated a positive anti‐double‐stranded DNA IgG antibody, supporting active lupus disease. Testing for human papillomavirus DNA and *Chlamydia trachomatis* was negative. Cervical cytology was negative for intraepithelial lesion or malignancy and showed reactive inflammatory changes.

Vulvar biopsy from a nonulcerated area demonstrated vacuolar interface dermatitis with follicular plugging and basement membrane thickening on routine hematoxylin and eosin staining. Special stains revealed stromal mucin deposition, a feature characteristic of DLE and not typically seen in lichen sclerosus (Figure 1A,B). Based on the clinical context and histopathologic findings, a diagnosis of vulvar hypertrophic DLE was made.

Figure 1(A) Periodic acid–Schiff stain demonstrating thickening of the basement membrane zone, a characteristic histopathologic feature of discoid lupus erythematosus. (B) Colloidal iron stain highlighting stromal mucin deposition within the dermis, supporting a diagnosis of discoid lupus erythematosus and distinguishing it from lichen sclerosus.

**Figure:**
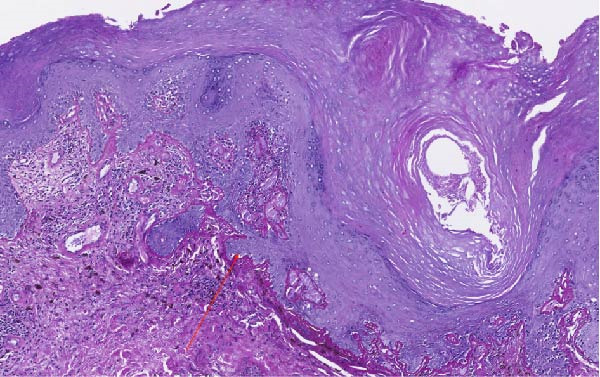
(A)

**Figure:**
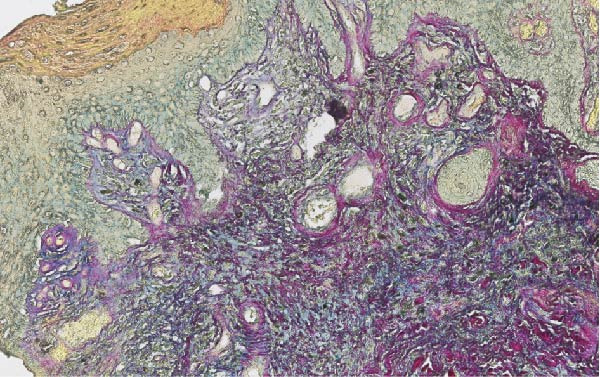
(B)

**Figure 2:**
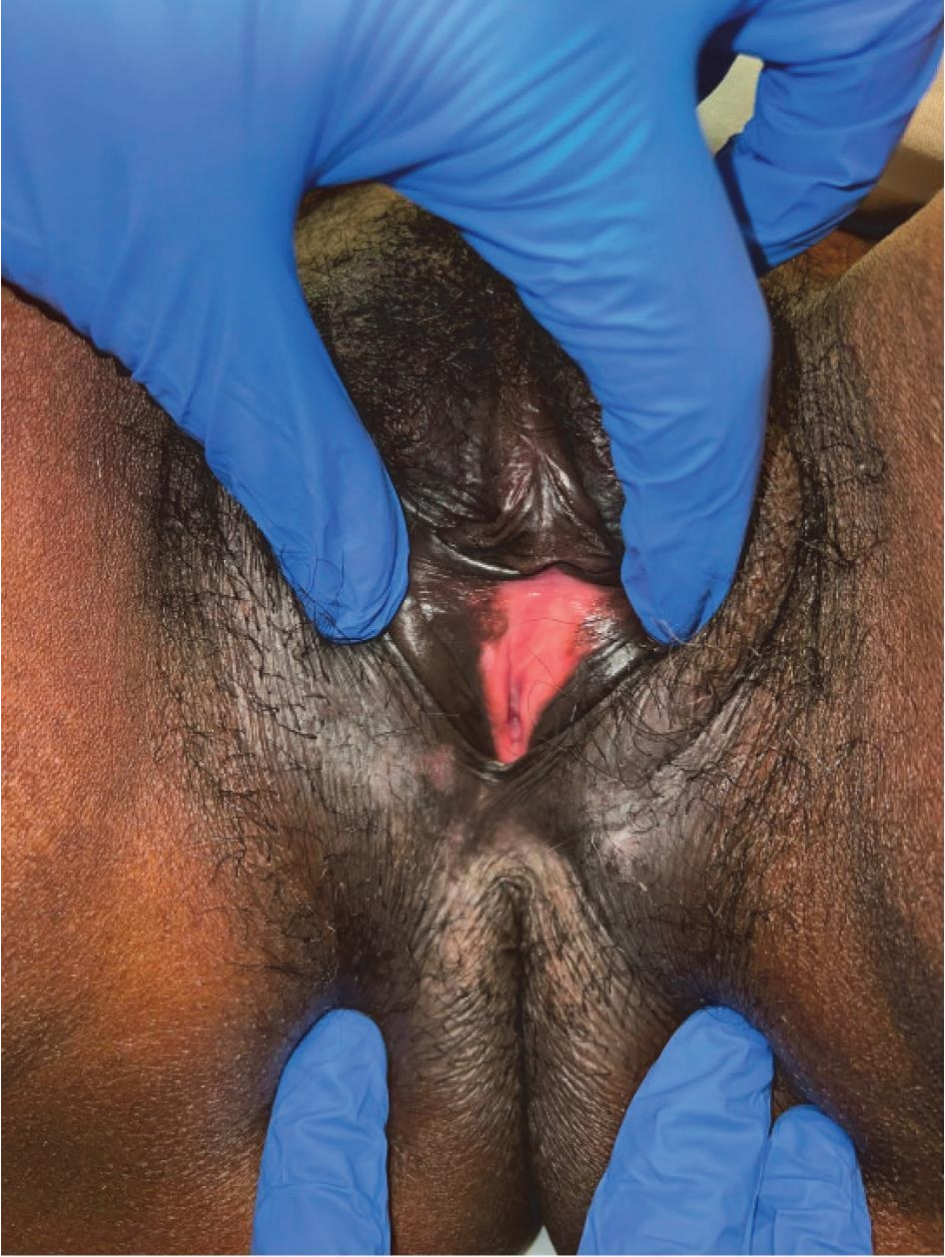
Clinical photograph of the vulva showing an erythematous, ulcerated plaque with residual postinflammatory hypopigmentation on follow‐up, consistent with the typical evolution of discoid lupus erythematosus lesions.

At follow‐up several weeks later, the lesion showed improvement with residual postinflammatory hypopigmentation (Figure 2). The patient was advised to continue treatment with hydroxychloroquine, azathioprine, and prednisone, with close rheumatologic follow‐up to guide corticosteroid tapering.

## 3. Discussion

Vulvar involvement in DLE is exceptionally rare and remains underrecognized in gynecologic practice. Fewer than 20 cases have been reported, most describing lesions initially misdiagnosed as more common vulvar dermatoses, including lichen sclerosus and herpes simplex virus infection [1, 2]. As highlighted in a recent review of vulvar autoimmune dermatoses, lupus affecting the vulva often presents diagnostic challenges due to overlapping clinical features and the absence of classic pruritus or pain [3].

Clinically, vulvar DLE may resemble lichen sclerosus through its hypopigmented or erythematous plaque‐like appearance. However, distinguishing features include an association with systemic lupus activity, relative absence of pruritus, and progression to postinflammatory dyspigmentation rather than architectural distortion. Histopathologic evaluation is essential, as routine hematoxylin and eosin staining may demonstrate interface dermatitis and basement membrane thickening seen in both conditions. The presence of stromal mucin, highlighted by special stains, favors a diagnosis of DLE and is not a feature of lichen sclerosus [1, 4–6].

## 4. Conclusion Statement

This case underscores the importance of maintaining a broad differential diagnosis for vulvar lesions, particularly in patients with known autoimmune disease. Timely biopsy and collaboration among gynecology, dermatology, pathology, and rheumatology are critical to establishing an accurate diagnosis and guiding appropriate immunosuppressive management. Increased awareness of vulvar DLE may improve diagnostic accuracy and prevent delays in care.

## Funding

No funding was received for this manuscript.

## Disclosure

All authors have read and approved the final version of the manuscript. The corresponding author had full access to all data associated with this case and takes responsibility for the integrity of the data and the accuracy of the report.

## Consent

Written informed consent was obtained from the patient for publication of this case report and the accompanying clinical images.

## Conflicts of Interest

The authors declare no conflicts of interest.

## Data Availability

Data sharing is not applicable to this article as no datasets were generated or analyzed during the current study.
